# A Novel Somatic Mutation of *CACNA1H* p.V1937M in Unilateral Primary Hyperaldosteronism

**DOI:** 10.3389/fendo.2022.816476

**Published:** 2022-06-09

**Authors:** Chi-Shin Tseng, Kang-Yung Peng, Shuo-Meng Wang, Yao-Chou Tsai, Kuo-How Huang, Wei-Chou Lin, Ya-Hui Hu, Vin-Cent Wu, Jeff S. Chueh

**Affiliations:** ^1^Graduate Institute of Clinical Medicine, National Taiwan University College of Medicine, Taipei, Taiwan; ^2^Department of Urology, National Taiwan University College of Medicine and Hospital, Taipei, Taiwan; ^3^Division of Nephrology, Department of Internal Medicine, National Taiwan University Hospital, Taipei, Taiwan; ^4^Division of Urology, Department of Surgery, Taipei Tzuchi Hospital, The Buddhist Tzu Chi Medical Foundation, New Taipei City, Taiwan; ^5^School of Medicine, Buddhist Tzu Chi University, Hualien, Taiwan; ^6^Department of Pathology, National Taiwan University Hospital, National Taiwan University College of Medicine, Taipei, Taiwan; ^7^Division of Endocrinology and Metabolism, Department of Internal Medicine, Taipei Tzu Chi Hospital, The Buddhist Medical Foundation, Taipei, Taiwan

**Keywords:** aldosterone producing adenoma, CACNA1H, primary aldosteronism, adrenalectomy, V1937M mutation

## Abstract

**Background:**

Somatic mutations for excess aldosterone production have been frequently identified as important roles in the pathogenesis of unilateral primary hyperaldosteronism (uPA). Although *CACNA1H* mutation represents a minor etiology in primary aldosteronism, it plays a significant role in causing uPAs in sporadic cases.

**Objective:**

To identify novel somatic *CACNA1H* mutation in patients with uPA and investigate the pathophysiological, immunohistological, and clinical characteristics of the variant.

**Methods:**

We applied a customized and targeted gene panel next-generation sequencing approach to detect mutations from the uPA cohort in Taiwan Primary Aldosteronism Investigation study group. Information from pre-diagnostic to postoperative data was collected, including past history, medications, blood pressure readings, biochemical data, and image studies. The functional role of the variant was confirmed by *in vitro* studies, demonstrating aldosterone production in variant-transfected human adrenal cell lines.

**Results:**

We identified a novel somatic *CACNA1H* mutation c.5809G>A (p.Val1937Met) in a uPA case. The *CACNA1H* gene encodes the pore-forming alpha-1H subunit of the voltage-dependent T-type calcium channel Cav3.2. This somatic *CACNA1H* p.V1937M variant showed excellent clinical and biochemical outcomes after ipsilateral adrenalectomy. The functional effect of somatic *CACNA1H* p.V1937M variant results in increased CYP11B2 expression and aldosterone biosynthesis in HAC15 cells. A distinct heterogeneous foamy pattern of CYP11B2 and CYP17A1 expression was identified in immunohistological staining, supporting the pathological evidence of aldosterone synthesis.

**Conclusions:**

The somatic mutation of *CACNA1H* p.V1937M might be a pathogenic driver in aldosterone overproduction. This study provides new insight into the molecular mechanism and disease outcomes of uPA.

## Introduction

Aldosterone-producing adenoma/nodule (APA/APN) is one of the common causes of primary aldosteronism (PA), while PA accounts for 5-10% of patients referred for evaluation of hypertension (1, 2). In patients with PA, the excessive production of plasma aldosterone results in a low concentration of plasma renin and leads to hypertension and hypokalemia (3). Several somatic mutations in *KCNJ5* (encoding potassium channel GIRK4, also known as Kir 3.4), *CACNA1D* (encoding α1 subunit of the Cav1.3 L-type voltage-dependent calcium channel), *ATP1A1* (encoding α1 subunit of the Na^+^/K^+^ ATPase), and *ATP2B3* (encoding plasma membrane Ca^2+^-transporting ATPase, type 3 PMCA3) genes, have been identified in at least 50% of patients with excess aldosterone production (4, 5). The abovementioned genetic mutations all increase intracellular calcium levels as a signal and, as a result, trigger the expression of *CYP11B2* to form aldosterone synthase, which catalyzes aldosterone biosynthesis (4–6).

Mutation in *CACNA1H* p.Met1549Val, encoding the pore-forming α1H subunit of the voltage-dependent T-type calcium channel Cav3.2, was found in children with early-onset PA *via* autosomal dominant transmission, as a cause of familial hyperaldosteronism (FH) type IV (7). Other gain-of-function germline *CACNA1H* variants, such as p.Met1549Ile, p.Ser196Leu, and p.Pro2083Leu, were also found in patients with FH (8). However, somatic mutations in *CACNA1H* have been scarcely identified in unilateral primary aldosteronism (uPA), because the large gene size of *CACNA1H* escalates the challenges of sequencing through conventional Sanger’s approach, which could only target selected exons or hotspot regions of the gene (9).

By using CYP11B2 immunohistochemistry (IHC)-guided targeted next-generation sequencing (NGS), aldosterone-driving somatic mutations were identified in over 90% of PAs (10). The detection of somatic mutations in uPA subtypes could allow physicians to highlight their distinct clinical and pathological features. Therefore, we performed customized and targeted gene panel NGS in patients with sporadic uPAs from Taiwan Primary Aldosteronism Investigation (TAIPAI) study group. The present study identified a novel somatic *CACNA1H* p.Val1937Met (V1937M) mutation in a patient with sporadic uPA. We further explored the pathophysiologic function of the classical uPA harboring *CACNA1H* variant on expressing aldosterone synthase.

## Materials and Methods

### Study Population

This study was approved by the Institutional Review Board of National Taiwan University Hospital (approval number 200611031R) and was conducted according to the principles of the Declaration of Helsinki. Written informed consent for clinical data collection, genomic analysis, and research use was obtained from the patient.

Patients with primary aldosteronism were detected and diagnosed by following the updated consensus of the Taiwan Society of Aldosteronism (11) and were enrolled in Taiwan Primary Aldosteronism Investigation (TAIPAI) study group with quality assurance from multiple institutions in Taiwan (12–17). Information from pre-diagnostic clinical data to postoperative outcomes was collected thoroughly and serially, including past history, medications, blood pressure readings, biochemical data, and image studies.

### Clinical Procedures

Patients with an abnormal plasma aldosterone to renin ratio (ARR) at screening were regarded as possible cases of PA (18), testing by commercial radioimmunoassay kits for plasma aldosterone concentration (PAC; ALDO-RIACT RIA kit, Cisbio Bioassays, Codolet, France) and plasma renin activity (PRA; Stillwater, MN, USA). Medications with the effect of suppressing or interfering with ARR were withheld before the tests for at least 3 weeks. Following the consensus of the Taiwan Society of Aldosteronism (11), a series of comprehensive studies for unilateral PA would be initiated as confirmatory tests and lateralization of aldosterone secretion. Lateralization of aldosterone secretion was confirmed *via* adrenal vein sampling (AVS) or dexamethasone suppression NP-59 SPECT/CT targeting adrenal tumor found on CT scan (19). Subsequently, ipsilateral adrenalectomy of the side of unilateral PA was performed in a lateral transperitoneal laparoscopic approach. Unilateral PA was further confirmed with a pathologically proven CYP11B2-positive stained adenoma or immunohistochemical evidence for multiple aldosterone-producing micronodules after adrenalectomy.

### Clinical and Biochemical Success

Patients were followed in the ambulatory office every month during the first three months after adrenalectomy and then every 3 months for at least one year. Their outcomes were measured by clinical and biochemical parameters according to the Primary Aldosteronism Surgery Outcome (PASO) criteria (20). Complete clinical success was defined as complete remission of hypertension without any antihypertensive medication at 12 months after surgery. Partial clinical success was defined as improved blood pressure with the same or less medication regimen or the same blood pressure with a reduction of medication. Complete biochemical success was defined as normal serum potassium levels and ARR. Partial biochemical success was defined as an improvement of hypokalemia and a decreased ARR (20).

### Tissue Immunohistochemistry

The formalin-fixed paraffin-embedded (FFPE) sections were blocked with 10% goat serum (Jackson ImmunoResearch Laboratories Inc., West Grove, PA, USA) for 1 hour at room temperature, then incubated overnight at 4°C with mouse monoclonal antibody for CYP11B2 and 17α-hydroxylase (CYP17A1) (generous gifts from Professor Celso Gomez-Sanchez) (21), and rat monoclonal antibody for CYP11B1 (MABS502, Sigma-Aldrich, USA). For detection of primary antibodies, HRP conjugated SignalStain^®^ Boost IHC Detection Reagent (Cell Signaling Technology, Danvers, MA, USA) was used (Vector Laboratories, Burlingame, USA). The sections were developed with the Liquid DAB+ Substrate Chromogen System (Dako, Agilent Technologies, Santa Clara, CA, USA) and counterstained with hematoxylin. Images were captured by using Olympus BX51 microscope with Olympus DP72 camera and processed using cell Sens Standard 1.14 software (Olympus, Hamburg, Germany).

### Nucleic Acid Extraction

Adrenal tumors were freshly preserved at −80°C. Genomic DNA was extracted from the adrenal tumor and peripheral whole blood. Genomic DNA from the adrenal tumor was extracted by using QIAamp DNA Mini Kit (Qiagen, Hilden, Germany); genomic DNA from whole blood was extracted by using Blood DNA Isolation Kit (Geneaid Biotech; New Taipei City, Taiwan) according to the instructions from manufacturers.

### Sequencing of the *CACNA1H* Gene

The coding sequence of *CACNA1H* gene was investigated by a customized and targeted gene panel next-generation sequencing approach, the targeted panel of aldosterone-driving genes including *KCNJ5, ATP1A1, ATP2B3, CACNA1D, CACNA1H, CLCN2*, and *CTNNB1*. The library construction contained amplicons targeting the full coding regions of these genes. PCR-based amplified products using Fluidigm Access-Array technology are followed by barcoding and next-generation resequencing on an Illumina MiSeq platform as previously reported (22).

The somatic *CACNA1H* p.V1937M variant was further confirmed by direct Sanger sequencing. The genomic DNA extracted from adrenal tumor and peripheral whole blood were sequenced following PCR amplification using specific primers: forward 5′-AAGCACTGCCTGAGCTAC-3′; reverse 5′- CATATCTTCCTGCTGGCTAC-3′. PCR was performed using GoTaq^®^ Green Master Mix (Promega, Madison, WI, USA). PCR cycling conditions were as follows: initial denaturation at 95°C for 5 minutes followed by 20 cycles of denaturation at 95 °C for 30 seconds, annealing at 52 °C for 30 seconds, and extension at 72 °C for 1 minute. This is followed by final extension at 72 °C for 7 minutes and the amplified product was stored at 4 °C. Direct sequencing of PCR products was performed using the BigDye Terminator version 3.1 cycle sequencing kit (Applied Biosystems, Foster City, USA) with a 3730 DNA Analyzer (Applied Biosystems, Foster City, CA, USA). Sequences were analyzed using DNAStar Lasergene SeqMan Pro 7.1.0 software

### Bioinformatics Analysis

For the predictions of sequence variants, computational (*in silico*) programs of Combined Annotation Dependent Depletion (CADD, v1.2) (23), Polymorphism Phenotyping v2 (PolyPhen-2 v2.2.2 build r394) (24), Sorting Intolerant From Tolerant (SIFT v1.03) (25), and the Mutation Significance Cutoff (MSC) generated by the CADD (MSC-CADD) (23) were applied. The combined prediction results, without failing more than one of the five *in silico* tools, would be considered as a single piece of evidence supporting a deleterious effect from a variant. Visualization of Cav3.2 Calcium Channel (CACNA1H) protein with the mutated sequence was performed by a Protter tool (26)

### Cell Culture

HAC15 human adrenocortical cells (27) were cultured in DMEM/F12 medium (Gibco; Thermo Fisher Scientific, Waltham, MA, USA) supplemented with ITS (Sigma-Aldrich, St. Louis, MO, USA), 1% penicillin–streptomycin and 10% FBS (Gibco; Thermo Fisher Scientific, Waltham, MA, USA) at 37°C in a humidified 5% CO2 incubator as previously reported (28, 29).

### Mutant *CACNA1H* Transfection and Treatment

The plasmids expressing the wild-type *CACNA1H* as well as *CACNA1H* p.V1937M mutation were constructed into the pIRES-EGFP-puro vector (Addgene plasmid #45567) using PCR-assisted, site-directed mutagenesis. We used PCR-based direct sequencing to confirm the mutation was successfully cloned into the vector. To evaluate the effect of mutant *CACNA1H* on the expression of CYP11B2 and aldosterone secretion, the HAC15 cells were transfected with pIRES-EGFP-wild-type *CACNA1H* or pIRES-EGFP *CACNA1H* p.V1937M using an Amaxa Nucleofector I (Lonza; 3 million cells, 3 µg of plasmid DNA; program X-005) according to the manufacturer’s instructions. The pIRES-EGFP empty vector was used as a control. After transfection, HAC15 cells were seeded at a density of 1 × 10^6^ cells/well into a 6-well plate. The cells were exposed to 10 nM angiotensin II (Sigma-Aldrich, St. Louis, MO, USA) 48 hours after transfection. The culture supernatant was collected 72 hours after transfection for measuring the concentrations of aldosterone, and cells were harvested for Western blot analysis.

### Western Blot Analysis

Whole-cell lysates were extracted by RIPA buffer (abcam, Cambridge, MA, USA) containing a protease inhibitor (Roche Diagnostics, Indianapolis, IN, USA). A 30 μg total protein was separated using 10% SDS-PAGE gels and electrophoretically transferred onto PVDF membranes. The membranes were then blocked for 1 hour at room temperature by using the BlockPRO™ blocking buffer (Visual Protein Biotechnology, Taipei, Taiwan) and then incubated overnight at 4°C with mouse monoclonal antibody for CYP11B2 (a kind gift from Professor Celso Gomez-Sanchez) at 1:1,000 dilution, and anti-GAPDH antibody (RRID : AB_10167668, Santa Cruz Biotechnology, Dallas, TX, USA) at 1:10,000 dilution in blocking buffer. After washing in Tris-buffered saline containing 0.1% Tween-20 (TBST) buffer, the membranes were incubated with HRP-conjugated anti-mouse secondary antibody (AB_330924, Cell Signaling Technology, Danvers, MA, USA) at 1:3,000 dilution or HRP-conjugated anti-rabbit secondary antibody (AB_2307391, Jackson Immuno-Research Laboratories, Westgrove, PA, USA) at 1:10,000 dilution for 1.5 hours at room temperature. Levels of proteins were detected by using the chemiluminescent detection reagents (Millipore, Billerica, MA, USA) which were used to detect the protein levels and visualized using a UVP Biospectrum 810 imaging system (Ultra Violet Products Ltd., Cambridge, UK). The intensity of each protein band was quantified by UVP software and normalized to GAPDH levels.

### Aldosterone Secretion

After HAC15 cells transfection with wild-type *CACNA1H* or p.V1937M mutation plasmid for 72 hours, the culture supernatant was collected to measure the aldosterone concentrations by using ALDO-RIACT RIA kit (Cisbio Bioassays, Codolet, France). Aldosterone levels were normalized against protein concentrations of cell lysates.

### Statistical Analysis

Experimental data were presented as mean ± standard error of mean (SEM) and the differences among groups with different transfected genes were analyzed by one-way ANOVA with *post-hoc* least significant difference (LSD) tests. Statistical analyses were performed with commercial statistical software SPSS (version 25.0; IBM Corp, SPSS, Inc, Chicago, IL, USA). A value of p < 0.05 was considered to indicate statistical significance.

## Results

### Identification of a Novel Somatic Cav3.2 Calcium Channel Variant

**Figure 1:**
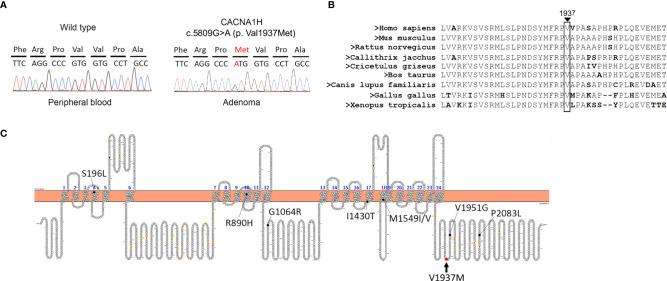
A somatic mutation in *CACNA1H* p.V1937M in aldosterone-producing nodule. **(A)** Results of DNA sequencing of *CACNA1H* in peripheral blood and aldosterone-producing nodule. *CACNA1H* p.V1937M mutation was noted in the nodule but not in blood cells. **(B)** The region marked by an arrow in wild-type *CACNA1H* indicates the location of valine (symbol Val or V) 1937. Amino acid V1937 is conserved across different species. **(C)** The structure of human *CACNA1H* Cav3.2 calcium channel. The red arrow indicates the replaced amino acid of the *CACNA1H* p.V1937M variant identified in this study, which is located in the C-terminal domain.

We performed a customized and targeted gene panel next-generation sequencing approach on DNA extracted from adrenal tumors for mutations which revealed one novel variant in *CACNA1H* c.5809G>A (p.Val1937Met) (**Table 1**). The sequence was changed by replacing a G with an A, resulting in a p.Val1937Met substitution in *CACNA1H*, which encodes the pore-forming alpha subunit of a T-type, low voltage-dependent calcium channel. This variant was only found in APN tissue but not in peripheral blood cells (**Figure 1A**). According to the evolutionary conservation analysis, amino acid V1937 in wild-type *CACNA1H* is conserved across different species (**Figure 1B**). The replaced amino acid of *CACNA1H* p.Val1937Met lies in the C-terminal domain close to the last transmembrane segment of the channel (**Figure 1C**).

**Table 1:** Novel CaV3.2 calcium channel mutation identified in uPA by next-generation sequencing.

Gene	mutation	Chr	Ref	Alt	Amino acid change (NM_021098.3)	ACMG Classification
CACNA1H	somatic	16	G	A	Val1937Met	likely pathogenic

Alt, alternative allele; Chr, chromosome; ACMG, American College of Medical Genetics and Genomics.

### Characteristics of the Identified Patient Harboring *CACNA1H* p.V1937M Variant

**Figure 2:**
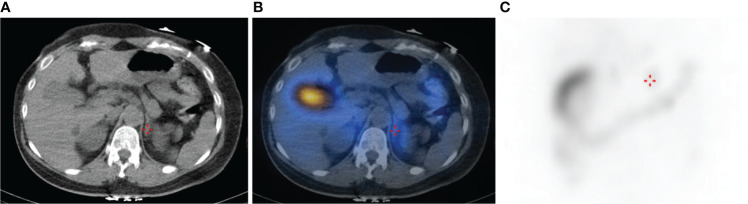
CT and NP-59 SPECT image of the abdomen showing a focus of increased tracer uptake in the region of the left adrenal gland (crosshairs). **(A)** Abdominal CT image of the axial plane shows a 0.6 cm left adrenal nodule. **(B)** Fused SPECT/CT image shows focal uptake at the left adrenal gland, indicating aldosterone-producing nodule. **(C)** Planar image shows mildly intense focal lesion over the left suprarenal area.

A novel somatic mutation of *CACNA1H* p.V1937M was identified in a classical uPA from a 43-year-old woman. She had persistent hypertension under medication control for more than 3 years and hypokalemia with serum potassium of 3.3 mmol/L. After a series of standard screening and confirmatory tests, PA was diagnosed. A computed tomography scan showed a 0.6 cm nodule over the left adrenal gland. Lateralization tests by 131I-labeled 6-β-iodomethyl-19-norcholesterol (NP-59) single photon-emission computed tomography (SPECT) and adrenal venous sampling both showed a hyperfunctioning left unilateral PA (**Figure 2**). She underwent laparoscopic left adrenalectomy uneventfully and achieved complete remission of hypokalemia and improved blood pressure with lower medication control. Her aldosterone level remained 104.19 ng/dL while plasma renin activity increased, resulting in a decreased ARR. Therefore, clinical and biochemical outcomes reached partial and complete success, respectively (**Table 2**).

**Table 2:** Clinical characteristics of the PA patient with a nodule harboring *CACNA1H* p.V1937M Variant.

Variables	*CACNA1H* p.V1937M
Age (years)	43
Sex	Female
Body weight (kg)	69.7
BMI (kg/m2)	27.7
CT mass size (cm)	Left: 0.6
NP-59	Hyperfunctioning left adrenal nodule
Prior to adrenalectomy	
Antihypertensive medications	Spironolactone 25mg twice daily Olmesartan 20mg once daily Amlodipine 5mg once daily
SBP (mmHg)	180
DBP (mmHg)	117
Aldosterone level (ng/dL)	101.02
PRA (ng/mL/hr)	1.48
ARR (ng/dL per ng/mL/h)	68.3
K (mEq/L)	3.3
12 months after adrenalectomy	
Antihypertensive medications	Amlodipine 5mg once daily
SBP (mmHg)	132
DBP (mmHg)	100
Aldosterone level (ng/dL)	104.19
PRA (ng/mL/hr)	4.57
ARR (ng/dL per ng/mL/h)	22.8
K (mEq/L)	4.1
Clinical success	Partial^1^
Biochemical success	Complete^2^

ARR, aldosterone renin ratio; BMI, body mass index; DBP, diastolic blood pressure; K, potassium; NP-59, The iodine-131 6-beta-iodomethyl-19-norcholesterol adrenal scintigraphy; PA, primary aldosteronism; PRA, plasma renin activity; SBP, systolic blood pressure. ^1^Partial clinical success: improved blood pressure with the same or less medication regimen or the same blood pressure with a reduction of medication. ^2^Complete biochemical success: a normal serum potassium levels and ARR at 12 months after adrenalectomy.

### The Histopathologic Features of Steroidogenic Enzymes in the Surgically Removed Adrenal With *CACNA1H* p.V1937M Variant

**Figure 3:**
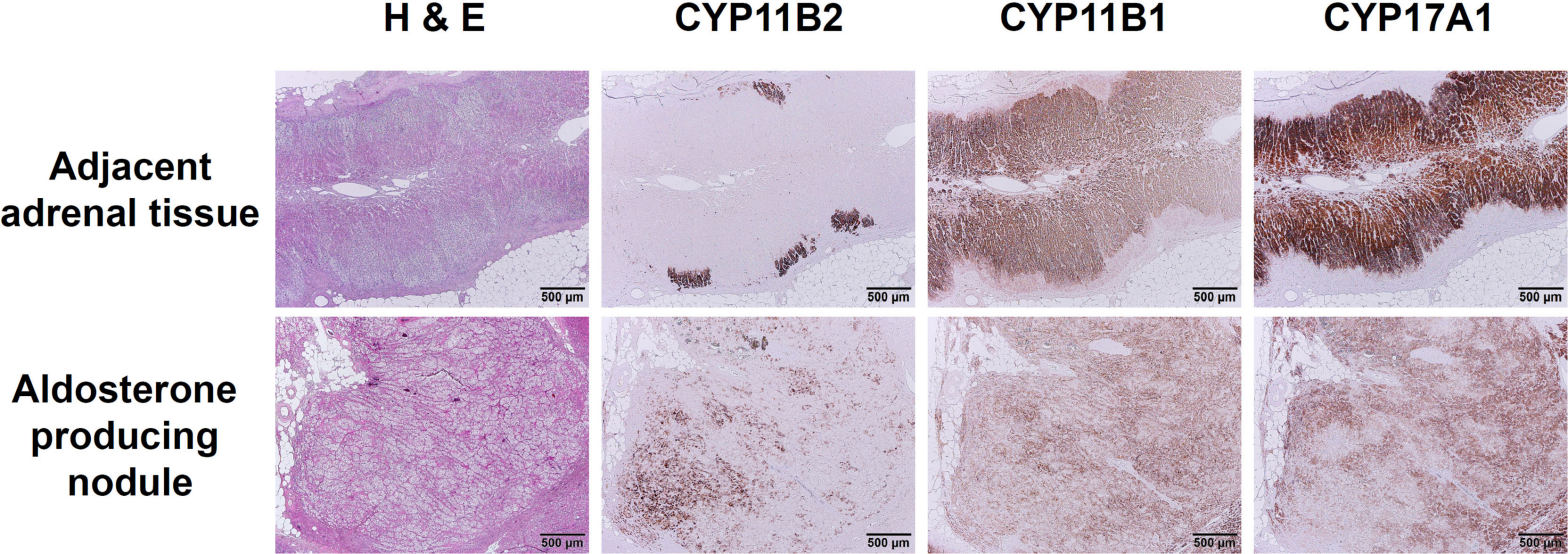
Immunohistochemical staining of CYP11B2, CYP11B1, and CYP17A1 in aldosterone-producing nodule with *CACNA1H* p.V1937M variant. The immunoreactivity of CYP11B2 IHC staining was prominent in *CACNA1H* p.V1937M variant nodule compared to the adjacent adrenal tissue with discrete aldosterone-producing micronodules. Little immunoreactivity of CYP11B1 and scattered immunoreactivity of CYP17A1 appeared in *CACNA1H* p.V1937M mutated nodule. Scale bar, 500 µm.

An aldosterone-producing nodule, composed of a mixture of clear cells and compact eosinophilic cells, was sectioned appropriately and could be distinguished from adjacent adrenal tissue by HE staining (**Figure 3**; H&E). In the nodule harboring *CACNA1H* p.V1937M variant, IHC staining of CYP11B2 showed a strong immunoreactivity and a heterogeneous foamy pattern, with the polarity of increasing immunoreactivity over the outer part of the lesion (**Figure 3**; CYP11B2). The adjacent adrenal tissue contained several aldosterone-producing micronodules with diffusely positive CYP11B2 immunostaining, which were morphologically indistinguishable by HE staining from the surrounding zona glomerulosa. The IHC staining of CYP11B1 and CYP17A1 were detectable in the adjacent adrenal tissue and aldosterone-producing nodule. The pattern of immunoreactivity in CYP11B1 and CYP17A1 IHC staining was more scattered in the nodule compared to the pattern in normal adjacent tissue (**Figure 3**; CYP11B1 and CYP17A1).

### Effect of *CACNA1H* p.V1937M Mutation on Expression of Aldosterone Synthase (CYP11B2) in Adrenal HAC15 Cells

**Figure 4:**
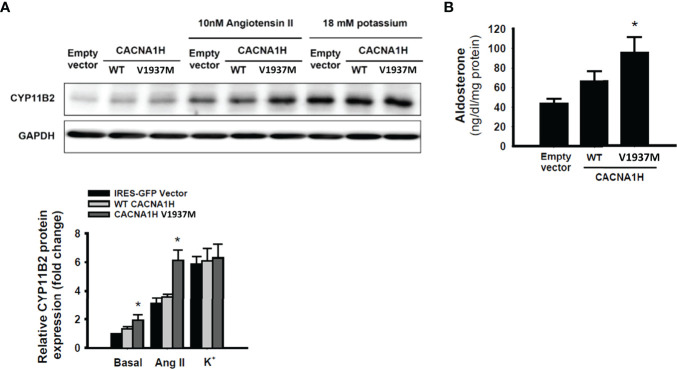
*CACNA1H* p.V1937M mutation increased CYP11B2 expression and aldosterone production. The HAC15 cells were transiently transfected with wild-type or mutant *CACNA1H* p.V1937M. The samples and culture medium were analyzed at 72 h after transfection. **(A)** The CYP11B2 protein expression in wild-type *CACNA1H* or *CACNA1H* p.V1937M transfected cells was determined in basal condition or stimulation with angiotensin II (10nM) or potassium (18mM). **(B)** The aldosterone production was higher in cells harboring *CACNA1H* p.V1937M mutant than either control or wild-type *CACNA1H* transfected cells under angiotensin II-stimulated condition. * indicated *P* < 0.05 vs wild-type KCNJ5 group.

To investigate the effect of *CACNA1H* p.V1937M mutation in adrenal cells, aldosterone biosynthesis was determined in adrenal HAC15 cells by transfection of wild-type *CACNA1H* or the *CACNA1H* p.V1937M variant. In basal condition, a significant increase of CYP11B2 expression was observed in HAC15 cells overexpressing *CACNA1H* p.V1937M compared to cells transfected with wild-type *CACNA1H* (**Figure 4A**). In the angiotensin II-stimulated condition, the expression of CYP11B2 was more prominent in HAC15 cells overexpressing *CACNA1H* p.V1937M compared to cells transfected with wild-type *CACNA1H* (**Figure 4A**). There was no significant difference in the expression of CYP11B2 between the *CACNA1H* p.V1937M and wild-type CACNA1H transfected cells under potassium stimulation (**Figure 4A**). The level of aldosterone secretion was significantly increased in culture supernatants of the cells transfected with *CACNA1H* p.V1937M mutant in angiotensin II-stimulated condition (**Figure 4B**).

## Discussion

In the present study, we identified a novel somatic mutation of *CACNA1H* p.V1937M in a classical uPA. Somatic mutations in genes coding for ion channels, such as *KCNJ5* and *CACNA1D*, and ATPases, such as *ATP1A1* and *ATP2B3* genes, have been widely reported in patients with PA (4, 5). However, somatic mutations in *CACNA1H* have been scarcely identified in uPA patients given their low prevalence and large gene size which increase the entire difficulty of sequencing (9). Through the conventional hot-spot Sanger sequencing approach, DNA was isolated from adrenal tumor tissue without targeting CYP11B2 expression, leading to a moderate detection rate of around 50-60% (22, 30). We have followed a customized and targeted gene panel NGS approach which could reach a close 80-90% detection rate of mutations in aldosterone-producing adenomas as a CYP11B2 IHC-guided targeted NGS approach (10, 31).

The NGS-derived variant of *CACNA1H* p.V1937M could be classified as likely pathogenic following the criteria of American College of Medical Genetics and Genomics guidelines (32). First, *in vitro* functional assays in the study have shown a gain-of-function effect of this missense variant on protein expression. Second, this patient’s phenotype of APN is highly specific for this genetic etiology, supporting the evidence of pathogenicity. Third, five *in silico* models (CADD, PolyPhen-2, SIFT, MSC-CADD, and MSC-PolyPhen2) were applied to aid in the impact of every presumed polymorphism, revealing damaging and functional consequences of this missense variant. Besides, sequence analysis of the patient’s germline DNA has confirmed this variant is truly somatic. Therefore, this novel somatic variant of *CACNA1H* p.V1937M could be considered a possible driver in patients with uPA.

Voltage-dependent T-type calcium channel α-subunit Cav3.2 is expressed mainly in adrenal gland zona glomerulosa (7). From the pathophysiological perspective, mutation of the Cav3.2 calcium channel encoded by *CACNA1H* leads to a clear gain-of-function effect of calcium signaling on aldosterone production and cell proliferation (33). This novel somatic mutation of *CACNA1H* p.V1937M was proven to increase CYP11B2 protein expression and aldosterone production in our *in vitro* functional studies in adrenal HAC15 cells with variant plasmid transfection. Mutations in the potassium channel encoded by *KCNJ5* lead to an increased Na^+^ conductance, depolarization of cells, and activation of calcium influx into the cell (34–36). Mutations in the calcium channel encoded by *CACNA1D* result in an increased Ca^2+^ influx into the cell. Mutations of two P-type ATPases, encoded by *ATP1A1*, and *ATP2B3*, can cause cell membrane depolarization and activate Ca^2+^ influx (37). Overall, the mechanism of PA in *KCNJ5* (5), *CACNA1D* (38), and *CACNA1H* p.V1937M variants all share the common final pathway by increasing calcium entry through the voltage-dependent channel.

Clinically, this patient with somatic mutation of *CACNA1H* p.V1937M presented with a substantially increased PAC of 101.02 ng/dl higher than other patients with uPA. This specific type of uPA along with a nodule was treated by surgical removal of the ipsilateral adrenal gland containing the adrenal nodule. She had complete biochemical success in achieving normal serum potassium levels and ARR after adrenalectomy. She achieved partial clinical success one year post-operatively with improved blood pressure and a reduced number of medications. Based on the evident improvement of biochemical and clinical parameters, along with the results from *in vitro* experiment, we infer that *CACNA1H* p.V1937M is a functional mutation.

The adrenal gland with *CACNA1H* p.V1937M somatic mutation showed a relatively small nodule with no zona glomerulosa hyperplasia. The nodule size was 0.6 cm in size which was identifiable on the CT scan. In contrast to somatic mutation, germline mutations can cause a wide range of adrenal hyperplasia. Germline mutations in *KCNJ5* G151R and T158A cause massive adrenal hyperplasia on CT scan whereas another mutation in *KCNJ5* G151E shows minimal hyperplasia (5, 39). Scholl et al. reported a germline mutation of *CACNA1H* p.M1549V which shows little or no hyperplasia by CT scan (7).

The age of PA onset of our case with *CACNA1H* p.V1937M mutation in the present study was around 40 years old. However, germline mutations, which cause less than 5% of PA, have been reported from patients with younger age and of the same or related genes as somatic mutations (40). *De novo* germline *CACNA1D* mutations were reported in children with PA, seizures, and neuromuscular abnormalities (38, 41). Germline mutations in *CLCN2*, encoding the voltage-dependent chloride channel protein ClC-2, were identified in familial hyperaldosteronism (FH) type II (42), while another somatic mutation in *CLCN2* was reported in a sporadic uPA (43). Different germline mutations in *KCNJ5* could be *de novo* or passed down in some families with different severity, known as FH type III (FH-III) (5, 44–46). Germline mutations in *CACNA1H* were found in FH as well. Five subjects with early-onset hypertension and germline mutation of *CACNA1H* p.M1549V were diagnosed with primary aldosteronism before age 10. The mutation of *CACNA1H* p.M1549V was transmitted through an autosomal dominant pattern in familial analysis and considered as FH type IV (7). Daniil et al. reported other germline *CACNA1H* variants which were associated with FH type II and with sporadic uPA. The variability of phenotypes in patients with different *CACNA1H* variants may result from different functional consequences by channel properties (8).

For IHC staining, the uPA with *CACNA1H* p.V1937M somatic mutation revealed a distinct pattern of heterogeneous immunoreactivity of CYP11B2 and CYP17A1 expression with foamy cells. The heterogeneity of CYP11B2 and CYP17A1 expression was observed in the somatic mutation of *CACNA1H* I1430T as well (9). The scattered pattern of CYP11B2 immunostaining implies multiple aldosterone-producing origins within a nodule. In contrast, aldosterone-producing micronodules in adjacent adrenal tissue contain a clustered pattern of CYP11B2 immunostaining. However, the mechanism of cell proliferation in *CACNA1H* mutated nodules requires further investigation in the future.

## Conclusions

In conclusion, we identified a novel somatic mutation of *CACNA1H* p.V1937M in a classical uPA. Our findings reveal a gain-of-function effect for *CACNA1H* in the regulation of CYP11B2 expression and adrenal aldosterone biosynthesis. Although somatic *CACNA1H* mutation represents a minor subset of APA/APN, it has a pathogenic role in causing uPA.

## Data Availability Statement

The datasets presented in this study can be found in online repositories. The names of the repository/repositories and accession number(s) can be found below: National Center for Biotechnology Information (NCBI) BioProject database under accession number SCV002062061.1.

## Ethics Statement

The studies involving human participants were reviewed and approved by the institutional review board of National Taiwan University Hospital. The patients/participants provided their written informed consent to participate in this study.

## Author Contributions

Conceptualization, C-ST, K-YP, and V-CW; methodology, K-YP; software, K-YP; validation, C-ST, K-YP, and V-CW; formal analysis, K-YP; investigation, W-CL, and V-CW; resources, S-MW, Y-CT, K-HH, W-CL, and V-CW; writing—original draft preparation, C-ST and K-YP; writing—review and editing, V-CW and JC; visualization, C-ST and K-YP; supervision, V-CW and JC; project administration, V-CW; funding acquisition, V.-C.W. All authors have read and agreed to the published version of the manuscript.

## Funding

This research was funded by the Ministry of Science and Technology, Taiwan, R.O.C. [MOST107-2314-B-002-026-MY3, 108-2314-B-002-058, 109-2314-B-002-174-MY3], National Health Research Institutes [PH-102-SP-09)], National Taiwan University Hospital [109-S4634, PC-1264, PC-1309, VN109-09, UN109-041, UN110-030], Grant MOHW110-TDU-B-212-124005 and Mrs. Hsiu-Chin Lee Kidney Research Fund, National Taiwan University New Faculty Grant (110L7460 and 111L7432), and Most 110-2314-B-002-239.

## Conflict of Interest

The authors declare that the research was conducted in the absence of any commercial or financial relationships that could be construed as a potential conflict of interest.

## Publisher’s Note

All claims expressed in this article are solely those of the authors and do not necessarily represent those of their affiliated organizations, or those of the publisher, the editors and the reviewers. Any product that may be evaluated in this article, or claim that may be made by its manufacturer, is not guaranteed or endorsed by the publisher.
