# Supplementary material for: A Novel Somatic Mutation of CACNA1H p.V1937M in Unilateral Primary Hyperaldosteronism
Source: Front Endocrinol (Lausanne). 2022 Jun 9;13:816476. doi: 10.3389/fendo.2022.816476 (PMC9218183; doi:10.3389/fendo.2022.816476)
Supplement: Supplementary file 1 [file Presentation_1.pdf]

Supplementary Material

Standard TAIPAI protocol and Aldosteronism Consensus in Taiwan

Patients were enrolled from the following hospitals:

There were 2 tertiary medical centers, 3 affiliated hospitals and 2 regional hospitals in various cities of Taiwan joining this investigator group(1). Patients with other secondary hypertension, including renovascular hypertension, Cushing's syndrome, hyperthyroidism, and pheochromocytoma were excluded from this study registry(2). All anti-hypertensive medications were discontinued for at least 21 days before screening tests. Doxazosin and/or diltiazem were administered to control markedly high blood pressure when required (3).

This study included two medical centers (National Taiwan University Hospital (NTUH), Taipei, Taiwan; Taipei University Hospital, Taipei, Taiwan) and five regional hospitals (Cardinal Tien Hospital, New Taipei City, Taiwan; Taipei Tzu Chi Hospital, New Taipei City, Taiwan; Yun- Lin Branch of NTUH, Douliou City, Taiwan; Hsin-Chu Branch of NTUH, Hsin-Chu City, Taiwan; Zhongxing Branch of Taipei City Hospital, Taipei, Taiwan)(4).

Our standard protocol to identify primary aldosteronism (PA) and functional lateralization:

The diagnosis of primary aldosteronism was established in hypertensive patients on the basis of the following criteria (5-7):

Confirmation

Fulfillment of the following three conditions confirmed a diagnosis of PA:

(1) autonomous excess aldosterone production evidenced with an aldosterone-renin ratio (ARR) > 35; (2) a TAIPAI score larger than 60% (8); (3) post-saline loading PAC > 16 ng/dL[#](9), or PAC/PRA > 35 (ng/dL)/(ng/mL/h) shown in a post-capotopril/losartan test .(10) (Abbreviations: PAC, plasma aldosterone concentration; PRA, plasma renin activity).

The probability of PA (TAIPAI score) was equal to:

$= 1 + e^{-\beta}$; where β = (PAC [ng/dl] × [0.063]) + PRA [ng/ml/h] × [−0.205]) + ([ARR × 0.001] BMI [kg/m^2] × [0.067]) + (Male × [−0.738] + SK [mmol/l] × [−1.512]) + (eGFR [ml/min/1.73 m^2] × [0.017]) + ([propensity score] × [−0.539] + [1.851]) (8)

#We used a criteria of autonomous excess aldosterone production evidenced with ARR > 35 ng/dL per ng/ml/hr after captopril test, TAIPAI score > 60%, (Wu, Yang et al. 2011) and post-saline

loading plasma aldosterone concentration (PAC) > 16 ng/dL to identify PA patients who received MRA treatment.

We have also done a systemic reviews of the performances of the saline infusion test. (table). The post saline loading aldosterone level was 14.5± 4.8ng/dL, while our criteria was within one standard deviation.

years	authors	Number of subjects	Post-test aldosterone threshold (pmol/L)	Post-test aldosterone threshold (ng/dL)	Diagnostic accuracy	Diagnostic criteria
2001	Agharazii M	44	246	19.07	Se 100%	CT/scintigraphy/AVS/surgery
2006	Giacchetti G	118	196	15.19	Se 88%Spe 100%	AVS/surgery outcome
2006	Mulatero P	98	139	10.78	Se 88%Spe 88%	FST positivity
2007	Rossi GP	120	196	15.19	Se 82%Spe 75%	CT/AVS
2012	Nanba K	57	170	13.18	Se 60%	Scintigraphy/AVS/surgery
2012	Willenberg HS	33	88	6.82	Se 82%Spe 92%	AVS/surgery outcome
2014	Ahmed AH	66	165 seated 140 supine	12.8 seated 10.9 supine	Se 96% seatedSe 33% supine	FST positivity
2016	Cornu E	199	139	10.78	29% of false negative	AVS/surgery outcome
2018	Song Y	236	222	17.21	Se 85%Spe 92%	FST positivity
2018	Meng X	164	310	24.03	Se 90.4%Spe 95.9%	AVS/Surgery outcome

Abbreviations: AVS, adrenal venous sampling; FST, Fludrocortisone suppression test; SIT, Saline infusion test;

Of note, not all patients received saline loading test and most of them received captopril test in our cohort (Wu, Chang et al. 2009). According to the consensus of Taiwan Aldosteronism Society(Wu, Hu et al. 2017), one positive confirmation test is considered adequate to make the diagnosis of primary aldosteronism. Compared with the criteria of other society that need one more confirmation study to diagnosis PA(Nishikawa, Omura et al. 2011), our survey criteria could have high sensitivity, not to say identified with high severity patients.

Outcome evaluation

Assessment of clinical and biochemical outcomes after unilateral adrenalectomy according to the PASO criteria. (15)

**OUTCOME**	**COMPLETE**<br>**(Remission)**<br>**Complete Cure**	**PARTIAL**<br>**(Improvement)**	**ABSENT**<br>**(Persistence)**
**CLINICAL SUCCESS**	Normal BP without antihypertensive medication	Same BP as before surgery with less antihypertensive medication<br><br>or decreased BP with the same or less antihypertensive medication	Unchanged or increased BP with the same or increased antihypertensive medication
**BIOCHEMICAL SUCCESS**	Correction of hypokalemia and normalization of ARR or suppression of aldosterone secretion in post-surgical confirmatory test<br><br>(if ARR elevated)	Correction of hypokalemia and elevated ARR with<br><br>≥ 50% reduction baseline PAC and/or elevated but improved post-surgical CCT	Persistent hypokalemia and/or persistent elevation of ARR with failure to suppress aldosterone secretion in post-surgical CCT

**INITIAL ASSESSMENT**	Initial outcome assessment within 3 months post-surgery for adjustment of anti-HT medication and correction of hypokalemia or hyperkaliemia if necessary
**FINAL ASSESSMENT**	Final outcome assessment at 6–12 months after adrenalectomy
**ASSESSMENT INTERVAL**	Reassessment of outcomes at yearly intervals for an indefinite period to exclude persistence or reoccurrence of disease

Abbreviations, ARR= aldosterone-to-renin ratio; BP= blood pressure; CCT=captopril challenging test; PAC= plasma aldosterone concentrations

References

1. C. H. Wu, Y. W. Yang, Y. H. Hu, Y. C. Tsai, K. L. Kuo, Y. H. Lin, S. C. Hung, V. C. Wu and K. D. Wu: Comparison of 24-h urinary aldosterone level and random urinary aldosterone-to-creatinine ratio in the diagnosis of primary aldosteronism. *PLoS One*, 8(6), e67417 (2013) doi:10.1371/journal.pone.0067417

2. S. Niizuma, H. Nakahama, K. Kamide, K. Fukuchi, Y. Iwanaga, H. Nakata, F. Yoshihara, T. Horio, S. Nakamura and Y. Kawano: The cutoff value of aldosterone-to-renin ratio for the diagnosis of primary aldosteronism in patients taking antihypertensive medicine. *Clin Exp Hypertens*, 30(7), 640-7 (2008) doi:10.1080/10641960802443282

3. K. D. Wu, T. S. Liao, Y. M. Chen, M. K. Lai, S. J. Chen, C. T. Su, T. S. Chu, C. C. Chang and B. S. Hsieh: Preoperative diagnosis and localization of aldosterone-producing adenoma by adrenal venous sampling after administration of metoclopramide. *J Formos Med Assoc*, 100(9), 598-603 (2001)

4. V. C. Wu, Y. H. Hu, C. H. Wu, C. C. Kao, C. Y. Wang, W. S. Yang, H. H. Lee, Y. S. Chang, Y. H. Lin, S. M. Wang, L. Chen, K. D. Wu and T. S. Group: Administrative data on diagnosis and mineralocorticoid receptor antagonist prescription identified patients with primary aldosteronism in Taiwan. *J Clin Epidemiol*, 67(10), 1139-49 (2014) doi:10.1016/j.jclinepi.2014.05.012

5. V. C. Wu, H. W. Chang, K. L. Liu, Y. H. Lin, S. C. Chueh, W. C. Lin, Y. L. Ho, J. W. Huang, C. K. Chiang, S. Y. Yang, Y. M. Chen, S. M. Wang, K. H. Huang, B. S. Hsieh and K. D. Wu: Primary Aldosteronism: Diagnostic Accuracy of the Losartan and Captopril Tests. *Am J Hypertens*, 22(8), 821-7 (2009)

6. C. C. Kuo, V. C. Wu, K. H. Huang, S. M. Wang, C. C. Chang, C. C. Lu, W. S. Yang, C. W. Tsai, C. F. Lai, T. Y. Lee, W. C. Lin, M. S. Wu, Y. H. Lin, T. S. Chu, C. Y. Lin, H. W. Chang, W. J. Wang, T. W. Kao, S. C. Chueh, K. D. Wu and G. Taipai Study: Verification and evaluation of

aldosteronism demographics in the Taiwan Primary Aldosteronism Investigation Group (TAIPAI Group). *J Renin Angiotensin Aldosterone Syst*, 12(3), 348-57 (2011) doi:10.1177/1470320310391329

7. V. C. Wu, C. C. Kuo, S. M. Wang, K. L. Liu, K. H. Huang, Y. H. Lin, T. S. Chu, H. W. Chang, C. Y. Lin, C. T. Tsai, L. Y. Lin, S. C. Chueh, T. W. Kao, Y. M. Chen, W. C. Chiang, T. J. Tsai, Y. L. Ho, S. L. Lin, W. J. Wang, K. D. Wu and T. S. Group: Primary aldosteronism: changes in cystatin C-based kidney filtration, proteinuria, and renal duplex indices with treatment. *J Hypertens*, 29(9), 1778-86 (2011) doi:10.1097/HJH.0b013e3283495cbb

8. V. C. Wu, S. Y. Yang, J. W. Lin, B. W. Cheng, C. C. Kuo, C. T. Tsai, T. S. Chu, K. H. Huang, S. M. Wang, Y. H. Lin, C. K. Chiang, H. W. Chang, C. Y. Lin, L. Y. Lin, J. S. Chiu, F. C. Hu, S. C. Chueh, Y. L. Ho, K. L. Liu, S. L. Lin, R. F. Yen, K. D. Wu and T. S. Group: Kidney impairment in primary aldosteronism. *Clin Chim Acta*, 412(15-16), 1319-25 (2011) doi:10.1016/j.cca.2011.02.018

9. C. H. Wu, V. C. Wu, Y. W. Yang, Y. H. Lin, S. Y. Yang, P. C. Lin, C. C. Chang, Y. C. Tsai, S. M. Wang and T. group: Plasma aldosterone after seated saline infusion test outperforms captopril test at predicting clinical outcomes after adrenalectomy for primary aldosteronism. *Am J Hypertens* (2019) doi:10.1093/ajh/hpz098

10. V. C. Wu, Y. H. Hu, L. K. Er, R. F. Yen, C. H. Chang, Y. L. Chang, C. C. Lu, C. C. Chang, J. H. Lin, Y. H. Lin, T. D. Wang, C. Y. Wang, S. T. Tu, S. C. Jeff Chueh, C. C. Chang, F. Y. Tseng, K. D. Wu and T. group: Case detection and diagnosis of primary aldosteronism - The consensus of Taiwan Society of Aldosteronism. *J Formos Med Assoc*, 116(12), 993-1005 (2017) doi:10.1016/j.jfma.2017.06.004

11. R. F. Yen, V. C. Wu, K. L. Liu, M. F. Cheng, Y. W. Wu, S. C. Chueh, W. C. Lin, K. D. Wu, K. Y. Tzen and C. C. Lu: 131I-6beta-iodomethyl-19-norcholesterol SPECT/CT for primary aldosteronism patients with inconclusive adrenal venous sampling and CT results. *J Nucl Med*, 50(10), 1631-7 (2009)

12. V. C. Wu, S. M. Wang, S. J. Chueh, S. Y. Yang, K. H. Huang, Y. H. Lin, J. J. Wang, R. Connolly, Y. H. Hu, C. E. Gomez-Sanchez, K. Y. Peng and K. D. Wu: The prevalence of CTNNB1 mutations in primary aldosteronism and consequences for clinical outcomes. *Sci Rep*, 7, 39121 (2017) doi:10.1038/srep39121

13. V. C. Wu, C. T. Chao, C. C. Kuo, Y. H. Lin, S. C. Chueh and K. D. Wu: Diagnosis and Management of Primary Aldosteronism. *Acta Nephrologica*, 26(3), 111-120 (2012)

14. C. C. Chang, B. C. Lee, Y. C. Chang, V. C. Wu, K. H. Huang, K. L. Liu and T. S. Group: Comparison of C-arm computed tomography and on-site quick cortisol assay for adrenal venous sampling: A retrospective study of 178 patients. *Eur Radiol*, 27(12), 5006-5014 (2017) doi:10.1007/s00330-017-4930-9

15. T. A. Williams, J. W. M. Lenders, P. Mulatero, J. Burrello, M. Rottenkolber, C. Adolf, F. Satoh, L. Amar, M. Quinkler, J. Deinum, F. Beuschlein, K. K. Kitamoto, U. Pham, R. Morimoto, H. Umakoshi, A. Prejbisz, T. Kocjan, M. Naruse, M. Stowasser, T. Nishikawa, W. F. Young, Jr., C. E. Gomez-Sanchez, J. W. Funder, M. Reincke and i. Primary Aldosteronism Surgery Outcome: Outcomes after adrenalectomy for unilateral primary aldosteronism: an international consensus on outcome measures and analysis of remission rates in an international cohort. *Lancet Diabetes Endocrinol*, 5(9), 689-699 (2017) doi:10.1016/S2213-8587(17)30135-3

frontiers
